# Data on docking of phytoconstituents of *Actinidia deliciosa* on dengue viral targets

**DOI:** 10.1016/j.dib.2019.103996

**Published:** 2019-05-17

**Authors:** Sneha R. Chandani, Kiran B. Lokhande, K. Venkateswara Swamy, Rabindra K. Nanda, Sohan S. Chitlange

**Affiliations:** a Dr. D. Y. Patil Unitech Society's, Dr. D. Y. Patil Institute of Pharmaceutical Sciences & Research, Pimpri, Pune 411018, India; b Bioinformatics Research Laboratory, Dr. D. Y. Patil Biotechnology and Bioinformatics Institute, Dr. D. Y. Patil Vidyapeeth, Pune 411033, India

**Keywords:** Docking, Actinidia, Dengue, Targets, Apigenin, Luteolin

## Abstract

Major Phytoconstituents of *Actinidia deliciosa* were explored for their anti-viral potential against dengue virus (DENV). The docking of these phytoconstituents was performed on 7 viral targets- 4 DENV non structural protein (NS5-SAM binding domain, NS5 RdRp domain, NS3 helicase & NS2B-NS3 protease) and 3 DENV structural proteins (Envelope protein-β-OD domain, stem domain & Domain III). The analysis was done on the basis of binding affinity, type of interactions (bond type and distance) and interaction with amino acids significant in viral replication. The top 5 phytoconstituents with best docking score have been reported.

**Table:** Specification table

Subject area	Computational and *insilico* Chemistry
More specific subject area	Docking studies
Type of data	Table, Image, Figure
How data was acquired	Molecular docking(Schrödinger Maestro Release 2016–4) (Schrödinger Release 2016–4**:** MS Jaguar, Schrödinger, LLC, New York, NY, 2016.), FlexX Lead IT 2.3.2
Data format	Raw & Analyzed
Experimental factors	Docking score and interaction with amino acid residues in the binding pocket
Experimental features	The structures of the 32 phytoconstituents were downloaded from Pubchem and energy minimized using Avogadro software. The minimized structures were docked on selected anti-viral targets using FlexX software.
Data source location	Pharmaceutical Chemistry Laboratory, Dr. D. Y. Patil Institute of Pharmaceutical Sciences & Research, Pimpri, Pune
Data accessibility	Data is with this article

**Table:** 

**Value of the data** • This is the first article which represents exploration of various phytoconstituents against several dengue virus targets. • It provides information about the phytoconstituents (belonging to varied classes) and their interaction with the viral targets which will help in investigating and developing other DENV inhibitors. • The data may be useful for researchers working on discovery and development of anti-dengue agents. • The promising phytoconstituents identified may serve as potential leads for development of future therapeutics for dengue infection.

## 1 Data

The phytoconstituent abundance, folklore usage and reported scientific literature of *Acitnidia deliciosa* develops a promising platform for its evaluation as antidengue agent [1], [2].

**Fig. 1:**
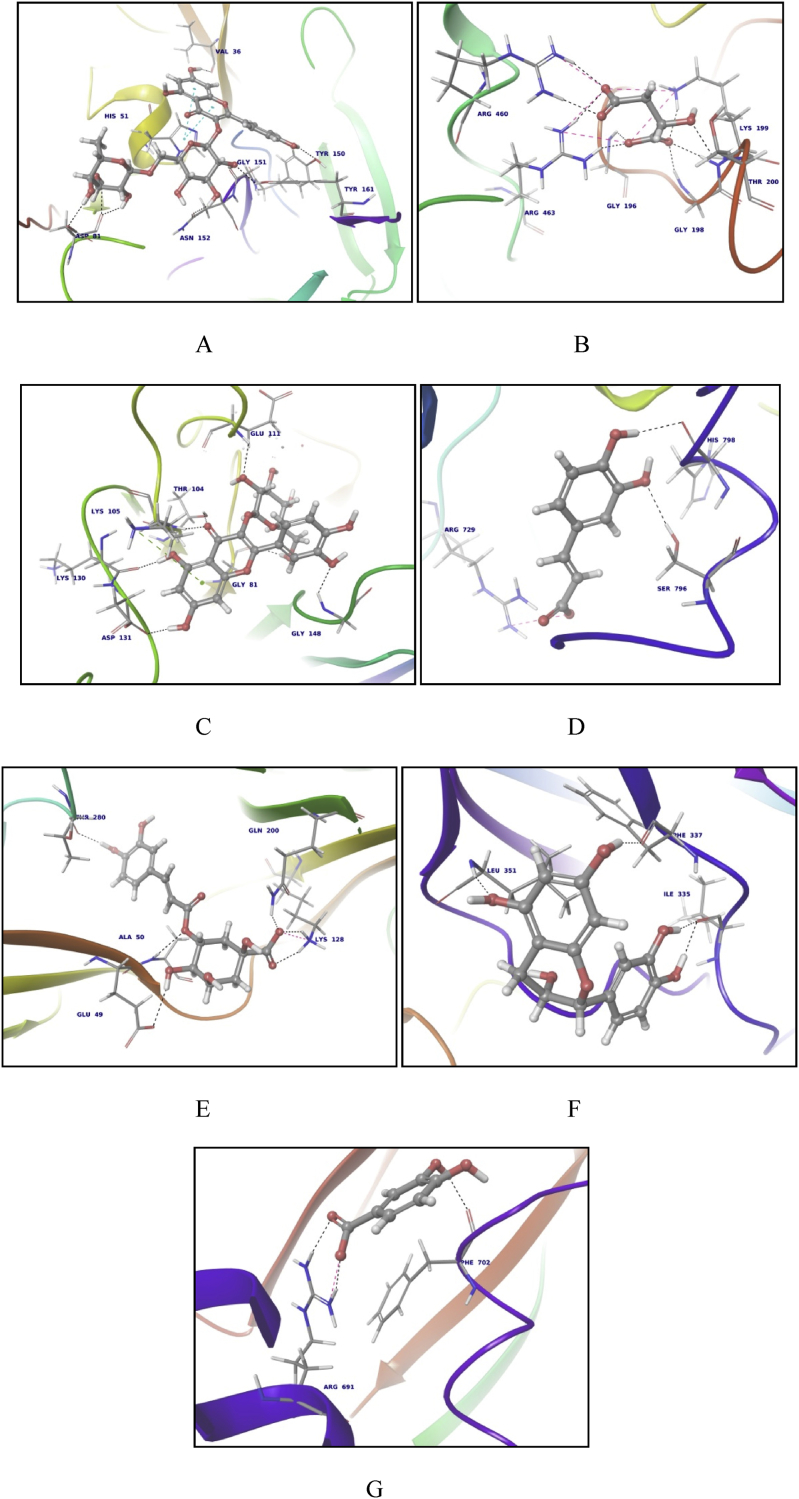
(All targets are shown as ribbon diagrams and phytoconstituents as ball and stick representation) A. 3UII – Kaempferol-3-rutinoside, B. 2JLV- Malic acid, C. 5EHI-Quercetin-3-glucoside, D. 5HMZ-Caffeic acid, E. IOKE-Chlorogenic acid, F. IOKE domain 3- Epicatechin, G. Stem Domain- Protocatechuic acid.

The data includes the outcome of docking of phytoconstituents *of Actinidia deliciosa* against various dengue viral targets. The details of viral targets (Table 1) and list of phytoconstituents (Table 2) are furnished. The top 5 best docked phytoconstituents are listed (Table 3) and image of the topmost phytoconstituent binding to selected viral proteins in provided (Fig. 1). The detail of docking of all phytoconstituents is provided in the supplementary data.

**Table 1:** Dengue viral targets used in docking.

Sr.No.	Protein	PDB ID	Resolution
1.	NS2B– NS3 protease	3UII [3]	2.3 Å
2.	NS3 helicase	2JLV [4]	1.9 Å
3.	NS5 (SAM Binding pocket)	5EHI [5]	1.303 Å
4.	NS5 (RdRp pocket)	5HMZ [6]	1.99 Å
5.	Envelope protein (hydrophobic pocket)	1OKE [7]	2.4 Å
6.	Envelope protein (binding domain 3)	1OKE [7]	2.4 Å
7.	Envelope protein (stem domain)	Model- UniProt B1PNV2[8], 3j27.1.A	–

**Table 2:** List of phytoconstituents.

1. Quercetin -3-glucoside 2. Kaempferol-3-rhamnoside 3. Epicatechin 4. Kaempferol-3-rutinoside 5. Riboflavine 6. Shikimic aicd 7. Caffeic acid 8. Chlorogenic acid 9. Protocatechuic acid 10. Quinic acid	11. Malic acid 12. Spermidine 13. Serotonin 14. p-coumaric acid 15. Niacin 16. Thiamine 17. Putrescine 18. Phylloquinone 19. Retinol 20. Tocomonoenol 21. δ-Tocopherol	22. Ascorbic acid 23. 3-Hydroxy-2-butanone 24. Phenylethyl alcohol 25. Ethyl-3-hydroxybutyrate 26. α-Terpineol 27. 23-Hydroxytormentic acid 28. 3-Methyl-2-butanol 29. Geraniol 30. 2-E-hexenal 31. α-Tocopherol 32. 9-cis-neoxanthin

**Table 3:** Top 5 phytoconstituents and their interactions.

**Sr. No.**	**Target**	**Name of the phytoconstituent**	**Binding energy (kCal/mol)**	**Interactions**	**Type of interaction**	**Bond distance (Å)**
1.	3UII	Kaempferol 3-rutinoside	−32.2516	Asp A:81	3 H-Bonds	1.811, 1.856, 2.408
				Asn B:152	H-Bond	2.509
				Tyr B:161	H-Bond	2.131
				Gly B:151	H-Bond	1.819
				Tyr B:150	H-Bond	2.153
				His B:51	Pi-Pi stacking	4.159
				Val B:36	H-Bond	1.636
		Riboflavin	−31.0863	Ser B:135	H-Bond	1.932
				Lys B:131	H-Bond	2.232
				Gly B:153	2 H-Bond	2.057, 2.21
				His B:51	Pi-Pi stacking	4.182
				TyrB:161	H-Bond	2.071
				Gly B:151	2 H-Bonds	1.783, 2.222
		Kaempferol 3-rhamnoside	−28.4051	His 51	H-Bond	1.837
				Lys 131	H-Bond	1.831
				Gly 151	2 H-Bonds	1.849, 2.178
				Tyr 161	H-Bond	2.32
				Gly 153	H-Bond	2.231
				Asn 152	H-Bond	2.251
		Quercetin 3-glucoside	−28.1772	His 51	Pi- Pi stacking	4.13
				Arg 54	H-Bond	2.634
				Asn 152	H-Bond	2.149
				Gly 153	H-Bond	1.883
				Tyr 161	H-Bond	1.73
				Gly 151	H-Bond	1.870
				Lys 131	H-Bond	2.374
				Tyr 150	H-Bond	2.075
				Val 36	H-Bond	2.190
		Chlorogenic acid	−25.997	Thr A:83	H-Bond	1.866
				Gly B:151	H-Bond	1.904
				Phe B:130	H-Bond	2.440
				Gly B:133	H-Bond	1.727
				Ser B:135	H-Bond	2.186
2.	2JLV	Malic acid	−50.8132	Gly 198	H-Bond	1.84
				Lys 199	H-Bond	1.92
					2 Salt bridges	4.19, 4.31
				Arg 463	2 H-Bonds	1.90, 2.29
				Gly 196	H-Bond	1.53
				Arg 460	H-Bond	2.04, 2.16
				Thr 200	H-Bond	2.14
		Ascorbic acid	−42.0454	Gln 456	H-Bond	1.75, 1.96
				Gly 196	H-Bond	2.06
				Ala 316	H-Bond	2.18
				Glu 285	H-Bond	2.12
				Thr 200	H-Bond	2.23
				Gly 198	2 H-Bonds	1.69, 2.07
				Lys 199	2 H-Bonds	1.89, 2.11
		Shikimic acid	−40.3817	Lys 199	H-Bond	1.95
					Salt bridge	4.85
				Arg 463	H-Bond	1.82
				Arg 460	H-Bond	2.39
				Gly 414	H-Bond	2.19
				Asn 416	H-Bond	2.00
				Thr 200	H-Bond	1.47
		Quinic acid	−37.6124	Glu 285	H-Bond	1.79
				Gly 414	H-Bond	2.27
				Thr 200	H-Bond	1.70
				Lys 199	Salt bridge	4.17
					H-Bond	1.90
				Gly 198	H-Bond	1.98
				Gly 196	H-Bond	1.47
		Protocatechuic acid	−37.3468	Asp 284	H-Bond	2.27
				Glu 285	H-Bond	2.06
				Ala 316	H-Bond	1.74
				Lys 199	Pi- cation	3.06
					Salt bridge	4.16
				Gly 196	H-Bond	1.99
				Arg 463	H-Bond	1.71
					H-Bond	1.71
				Arg 460	H-Bond	2.22
3.	5EHI	Quercetin 3-glucoside	−32.7547	Gly 148	H-Bond	1.99
				Glu 111	H-Bond	2.28
				Asp 131	H-Bond	1.58
				Gly 81	H-Bond	2.2
				Lys 105	Pi-cation	6.13
					H-Bond	1.61
				Lys 130	H-Bond	1.73
				Thr 104	H-Bond	2.23
		Kaempferol 3-rhamnoside	−31.1157	Gly 148	H-Bond	1.78
				Thr 104	H-Bond	1.87
				Lys 105	H-Bond	2.13
				Lys 130	H-Bond	2.11
				Asp 131	H-Bond	1.51
		Epicatechin	−28.6107	Lys 130	H-Bond	1.78
				Val 132	2 H-Bonds	1.70, 2.33
				Asp 131	H-Bond	2.29
				Gly 148	H-Bond	1.81
				Asp 146	H-Bond	2.21
				Gly 81	H-Bond	1.81
		Ascorbic acid	−26.2121	Asp 146	2 H-Bonds	1.84, 1.91
				Ser 56	H-Bond	2.23
				Gly 85	H-Bond	2.35
				Gly 86	H-Bond	1.83
				Trp 87	H-Bond	2.27
		Kaempferol 3-rutinoside	−24.9142	Glu 111	H-Bond	1.73
				Thr 104	H-Bond	2.20
				Lys 130	H-Bond	2.13
				Lys 105	H-Bond	2.09
					Pi cation	6.35
4.	5HMZ	Caffeic acid	−24.8427	His 798	H-Bond	2.07
				Ser 796	H-Bond	2.3
				Arg 729	Salt bridge	3.74
		Epicatechin	−24.3502	His 798	H-Bond	2.33
				Thr 794	H-Bond	2.08
				Ser 710	H-Bond	1.95
		Chlorogenic acid	−23.9068	Leu 511	H-Bond	2.43
				Arg 729	3 H-Bonds	1.97, 1.99, 2.73
				Ser 710	H-Bond	2.18
		Quinic acid	−22.7297	Arg 729	2 H-Bonds	1.85, 2.01
				Trp 795	H-Bond	2.13
		Kaempferol 3-rhamnoside	−22.0444	Thr 794	H-Bond	1.74
				Tyr 766	H-Bond	2.37
				Thr 793	H-Bond	1.97
				Ser 710	H-Bond	2.29
				Arg 729	Pi cation	5.71
5.	1OKE	Chlorogenic acid	−21.3989	Lys 128	2 H-Bonds	1.73, 2.08
				Thr 280	H-Bond	1.8
				Gln 200	H-Bond	1.85
				Ala 50	H-Bond	2.36
				Glu 49	H-Bond	2.26
		Serotonin	−20.906	Thr 48	H-Bond	1.63
				Thr 280	H-Bond	1.62
		Epicatechin	−19.2868	Thr 48	H-Bond	1.99
				Phe 279	H-Bond	2.21
				Thr 280	H-Bond	2.06
		Quercetin 3-glucoside	−19.1458	Gln 271	H-Bond	2.43
				Thr 48	H-Bond	2.04
				Glu 49	H-Bond	1.82
				Ala 50	H-Bond	1.48
		Caffeic acid	−18.9577	Thr 48	2 H-Bonds	1.83, 1.92
				Ala 50	H-Bond	1.94
				Lys 128	Salt bridge	2.69
6.	1OKE- Domain III	Epicatechin	−20.691	Ile 335	2 H-Bonds	1.73, 1.78
				Phe 337	H-Bond	2.20
				Leu 351	H-Bond	1.89
		Kaempferol 3-rhamnoside	−14.6036	Phe 337	H-Bond	2.08
				Gly 381	H-Bond	1.90
		Protocatechuic acid	−14.4177	Phe 337	H-Bond	1.93
				Pro 356	H-Bond	1.93
				Asn 355	H-Bond	1.88
		Serotonin	−14.2435	Glu 338	Salt bridge	3.77
				Ile 335	H-Bond	1.54
				Asn 355	H-Bond	2.17
		Shikimic acid	−13.6214	Asn 355	2 H-Bonds	2.06, 2.08
				Phe 337	H-Bond	2.01
7.	Model- Uniprot B1PNV2, 3j27.1.A	Protocatechuic acid	−19.4906	Arg 691	2 H-Bonds	1.65, 1.89
					Salt bridge	2.61
				Phe 702	H-Bond	1.80
		Shikimic acid	−18.5522	Arg 691	H-Bond	1.81
					Salt bridge	2.88
				Phe 702	H-Bond	2.12
		p-coumaric acid	−18.4392	Arg 691	2 H-Bonds	1.52, 2.03
		Caffeic acid	−17.9774	Arg 691	H-Bond	1.74
					Salt bridge	2.85
				Trp 700	H-Bond	1.79
		Malic acid	−17.3804	Arg 691	2 H-Bonds	1.94, 1.98
					Salt bridge	4.08

## 2 Experimental design, materials, and methods

### 2.1 Selection & retrieval of target structures

The selection of targets was done on the basis of literature survey. The docking was carried out on 4 non structural (NS2B-NS3, NS3 helicase, NS5 methyltransferase, NS5 RdRp domain) proteins and 3 possible target sites in envelope protein of the dengue virus.

The crystal structures of the selected dengue non structural proteins and envelope protein were retrieved from protein data bank. (PDB database, www.rcsb.org). The downloaded protein structure was prepared prior to docking using Schrödinger Maestro release 2016–4. Briefly the protein preparation was done by preprocessing the structures for assignment of bonds and bond orders, addition of hydrogens, filling in missing loops or side chains, capping uncapped termini, adjusting bonds and formal charges for metals, and correcting mislabeled elements, removing water molecules, removing unwanted chains and optimization of hydrogen bonded structures followed by refinement.

The Table 1 gives details of X-ray crystallographic models of targets used for docking.

No suitable protein structure was available in protein data bank for stem domain of the envelope protein. A model was created using dengue full length dengue envelope protein (UniProt ID- B1PNV2_9FLAV) with SWISS-MODEL [9].

The receptor preparation was done using Schrodinger Maestro Protein preparation wizard.

### 2.2 Ligand preparation and molecular docking

The structures of the selected 32 phytoconstituents of *Actinidia deliciosa* were downloaded from Pubchem (https://pubchem.ncbi.nlm.nih.gov/). The energy minimisation was done using Avogadro software and structures were saved in sdf format. The minimized structures were docked on the prepared protein targets after converting to 3d structures and refinement, using FlexX Lead IT 2.3.2 software.

The best phytoconstituent was identified on the basis of binding energy and interaction with amino acid residues important for viral replication. Table 3 gives the top 5 phytoconstituents based on binding energy and Fig. 1 gives the best docked phytoconstituent per dengue viral target.
