# Supplementary material for: Data on docking of phytoconstituents of Actinidia deliciosa on dengue viral targets
Source: Data Brief. 2019 May 17;25:103996. doi: 10.1016/j.dib.2019.103996 (PMC6626881; doi:10.1016/j.dib.2019.103996)
Supplement: Multimedia component 1 [file mmc1.docx]

Conflict of Interest: NONE
