# Supplementary material for: Data on docking of phytoconstituents of Actinidia deliciosa on dengue viral targets
Source: Data Brief. 2019 May 17;25:103996. doi: 10.1016/j.dib.2019.103996 (PMC6626881; doi:10.1016/j.dib.2019.103996)
Supplement: Supplementary file 2 — Multimedia component 2 [file mmc2.docx]

**Interaction of *Actinidia deliciosa* phytoconstituents to various viral targets**

**Target- NS3 Helicase ( PDB ID- 2JLV)**

| **Sr. No.** | **Ligand** | **Dock Score** | **Interacting Residue** | **Bond Type** | **Bond distance** |
| --- | --- | --- | --- | --- | --- |
|  | **Ascorbic acid** | **-42.045** | **Gln 456** | **H-Bond(Sidechain)** | **1.75** |
|  |  |  |  | **H-Bond(Sidechain)** | **1.96** |
|  |  |  | **Ala 316** | **H-Bond(Backbone)** | **2.18** |
|  |  |  | **Glu 285** | **H-Bond(Sidechain)** | **2.12** |
|  |  |  | **Thr 200** | **H-Bond(Sidechain)** | **2.23** |
|  |  |  | **Gly 198** | **H-Bond(Backbone)** | **1.69** |
|  |  |  |  | **H-Bond(Backbone)** | **2.07** |
|  |  |  | **Lys 199** | **H-Bond(Backbone)** | **2.11** |
|  |  |  |  | **H-Bond(Sidechain)** | **1.89** |
|  |  |  | **Gly 196** | **H-Bond(Backbone)** | **2.06** |
|  | **Ethyl-3-hydorxy-butyrate** | **-25.933** | **Thr 200** | **H-Bond(Backbone)** | **1.92** |
|  |  |  | **Gly 198** | **H-Bond(Backbone)** | **1.98** |
|  |  |  | **Lys 199** | **H-Bond(Backbone)** | **1.90** |
|  |  |  | **Arg 463** | **H-Bond(Sidechain)** | **2.09** |
|  | **3-hydroxy-2-butanone** | **-23.49** | **Lys 199** | **H-Bond(Backbone)** | **2.11** |
|  |  |  | **Gly 196** | **H-Bond(Backbone)** | **1.76** |
|  |  |  | **Arg 463** | **H-Bond(Sidechain)** | **1.75** |
|  | **3-methyl-2-butanone** | **-14.232** | **Lys 199** | **H-Bond(Backbone)** | **1.93** |
|  |  |  | **Gly 198** | **H-Bond(Backbone)** | **1.89** |
|  | **Geraniol** | **-10.4018** | **Lys 199** | **H-Bond(Sidechain)** | **1.87** |
|  | **2- E-hexenal** | **-8.4822** | **Ala 316** | **H-Bond(Backbone)** | **2.06** |
|  |  |  | **Gln 456** | **H-Bond(Sidechain)** | **1.89** |
|  | **α-Terpineol** | **-6.9091** | **Ala 197** | **H-Bond(Backbone)** | **1.85** |
|  | **Phenylethylalcohol** | **-5.9628** | **Lys 199** | **H-Bond(Sidechain)** | **1.73** |
|  | **23-hydroxytormentic acid** | **0.9022** | **Arg 418** | **H-Bond(Sidechain)** | **1.85** |
|  |  |  | **Asn 416** | **H-Bond(Backbone)** | **2.50** |
|  |  |  | **Lys 201** | **H-Bond(Sidechain)** | **1.95** |
|  | **Malic acid** | **-50.8132** | **Gly 198** | **H-Bond(Backbone)** | **1.84** |
|  |  |  | **Lys 199** | **H-Bond(Backbone)** | **1.92** |
|  |  |  |  | **2 Salt bridge** | **4.19, 4.31** |
|  |  |  | **Arg 463** | **H-Bond(Sidechain)** | **1.90** |
|  |  |  |  | **H-Bond(Sidechain)** | **2.29** |
|  |  |  |  | **2 Salt bridge** | **3.26, 3.62** |
|  |  |  | **Gly 196** | **H-Bond(Backbone)** | **1.53** |
|  |  |  | **Arg 460** | **H-Bond(Sidechain)** | **2.16** |
|  |  |  |  | **H-Bond(Sidechain)** | **2.04** |
|  |  |  |  | **Salt bridge** | **3.15** |
|  |  |  | **Thr 200** | **H-Bond(Backbone)** | **2.14** |
|  | **Shikimic acid** | **-40.3817** | **Lys 199** | **H-Bond(Sidechain)** | **1.95** |
|  |  |  |  | **Salt bridge** | **4.85** |
|  |  |  | **Arg 463** | **H-Bond(Sidechain)** | **1.82** |
|  |  |  | **Arg 460** | **H-Bond(Sidechain)** | **2.39** |
|  |  |  | **Gly 414** | **H-Bond(Backbone)** | **2.19** |
|  |  |  | **Asn 416** | **H-Bond(Sidechain)** | **2.00** |
|  |  |  | **Thr 200** | **H-Bond(Backbone)** | **1.47** |
|  | **Quinic acid** | **-37.6124** | **Glu 285** | **H-Bond(Sidechain)** | **1.79** |
|  |  |  | **Gly 414** | **H-Bond(Backbone)** | **2.27** |
|  |  |  | **Thr 200** | **H-Bond(Backbone)** | **1.70** |
|  |  |  | **Lys 199** | **Salt bridge** | **4.17** |
|  |  |  |  | **H-Bond(Backbone)** | **1.90** |
|  |  |  | **Gly 198** | **H-Bond(Backbone)** | **1.98** |
|  |  |  | **Gly 196** | **H-Bond(Backbone)** | **1.47** |
|  | **Protocatechuic acid** | **-37.3468** | **Asp 284** | **H-Bond(Sidechain)** | **2.27** |
|  |  |  | **Glu 285** | **H-Bond(Sidechain)** | **2.06** |
|  |  |  | **Ala 316** | **H-Bond(Backbone)** | **1.74** |
|  |  |  | **Lys 199** | **Pi- cation** | **3.06** |
|  |  |  |  | **Salt bridge** | **4.16** |
|  |  |  | **Gly 196** | **H-Bond(Backbone)** | **1.99** |
|  |  |  | **Arg 463** | **H-Bond(Sidechain)** | **1.71** |
|  |  |  |  | **H-Bond(Sidechain)** | **1.71** |
|  |  |  | **Arg 460** | **H-Bond(Sidechain)** | **2.22** |
|  | **Niacin** | **-35.6719** | **Arg 463** | **Salt bridge** | **4.33** |
|  |  |  | **Lys 199** | **H-Bond(Sidechain)** | **2.16** |
|  |  |  | **Arg 460** | **Salt bridge** | **4.15** |
|  |  |  |  | **H-Bond(Sidechain)** | **2.18** |
|  | **Kaemferol-3-rutinoside** | **-32.0556** | **Arg 463** | **H-Bond(Backbone)** | **2.21** |
|  |  |  | **Gly 196** | **H-Bond(Backbone)** | **1.80** |
|  |  |  |  | **H-Bond(Backbone)** | **1.44** |
|  |  |  | **Asn 416** | **H-Bond(Backbone)** | **1.26** |
|  |  |  |  | **H-Bond(Sidechain)** | **2.16** |
|  |  |  |  | **H-Bond(Sidechain)** | **1.99** |
|  |  |  | **Gly 414** | **H-Bond(Backbone)** | **1.87** |
|  | **Caffeic acid** | **-31.1352** | **Gly 198** | **H-Bond(Backbone)** | **1.92** |
|  |  |  | **Lys 199** | **H-Bond(Backbone)** | **2.02** |
|  |  |  |  | **Salt bridge** | **3.93** |
|  |  |  | **Arg 463** | **H-Bond(Sidechain)** | **2.08** |
|  |  |  | **Asn 416** | **H-Bond(Sidechain)** | **1.81** |
|  | **p-coumaric acid** | **-27.3511** | **Lys 201** | **Pi- cation** | **5.95** |
|  |  |  | **Lys 199** | **H-Bond(Backbone)** | **2.19** |
|  |  |  |  | **Salt bridge** | **4.09** |
|  |  |  | **Arg 463** | **H-Bond(Sidechain)** | **1.79** |
|  |  |  | **Gly 196** | **H-Bond(Backbone)** | **1.69** |
|  | **Chlorogenic acid** | **-23.8202** | **Lys 199** | **H-Bond(Backbone)** | **1.96** |
|  |  |  | **Gly 198** | **H-Bond(Backbone)** | **1.54** |
|  |  |  | **Arg 463** | **Pi- cation** | **5.39** |
|  |  |  |  | **Pi Pi stacking** | **4.72** |
|  |  |  | **Lys 201** | **H-Bond(Sidechain)** | **2.11** |
|  |  |  |  | **Salt bridge** | **1.95** |
|  |  |  | **Lys 398** | **Salt bridge** | **3.12** |
|  |  |  | **Asn 416** | **H-Bond(Backbone)** | **2.29** |
|  | **Quercetin-3-glucoside** | **-23.2571** | **Arg 463** | **H-Bond(Backbone)** | **2.09** |
|  |  |  |  | **H-Bond(Backbone)** | **2.43** |
|  |  |  |  | **Pi Pi stacking** | **4.49** |
|  |  |  |  | **Pi cation** | **5.00** |
|  |  |  | **Gly 198** | **H-Bond(Backbone)** | **1.77** |
|  |  |  | **Lys 199** | **H-Bond(Backbone)** | **2.15** |
|  |  |  | **Gly 196** | **H-Bond(Backbone)** | **1.38** |
|  |  |  | **Lys 201** | **H-Bond(Sidechain)** | **2.45** |
|  |  |  | **Arg 418** | **Pi cation** | **5.77** |
|  |  |  | **Asn 416** | **H-Bond(Sidechain)** | **2.14** |
|  |  |  | **Thr 200** | **H-Bond(Sidechain)** | **1.96** |
|  | **Epicatechin** | **-21.6963** | **Glu 285** | **H-Bond(Sidechain)** | **1.63** |
|  |  |  |  | **H-Bond(Backbone)** | **2.39** |
|  |  |  | **Ala 316** | **H-Bond(Backbone)** | **1.97** |
|  |  |  | **Lys 199** | **Pi cation** | **3.01** |
|  |  |  | **Lys 201** | **H-Bond(Backbone)** | **1.61** |
|  |  |  | **Arg 463** | **Pi Pi stacking** | **5.06** |
|  |  |  | **Arg 460** | **H-Bond(Sidechain)** | **1.79** |
|  | **Kaempferol -3-rhamnoside** | **-20.903** | **Lys 199** | **H-Bond(Backbone)** | **2.02** |
|  |  |  | **Gly 198** | **H-Bond(Backbone)** | **1.88** |
|  |  |  | **Arg 463** | **Pi Pi stacking** | **4.61** |
|  |  |  |  | **Pi- cation** | **5.13** |
|  |  |  | **Thr 200** | **H-Bond(Sidechain)** | **1.51** |
|  |  |  | **Asn 416** | **H-Bond(Sidechain)** | **2.13** |
|  |  |  | **Lys 201** | **Pi- cation** | **5.11** |
|  |  |  | **Gly 196** | **H-Bond(Backbone)** | **2.00** |
|  |  |  | **Ala 197** | **H-Bond(Backbone)** | **1.94** |
|  | **Serotonin** | **-17.792** | **Arg 463** | **2 Pi- cation** | **4.44, 4.73** |
|  |  |  |  | **2 Pi Pi stacking** | **4.66, 4.33** |
|  |  |  | **Gly 414** | **H-Bond(Backbone)** | **1.66** |
|  | **Putrescine** | **-17.1229** | **Glu 285** | **H-Bond(Sidechain)** | **1.45** |
|  |  |  | **Asp 284** | **H-Bond(Sidechain)** | **1.81** |
|  |  |  | **Gln 456** | **H-Bond(Sidechain)** | **1.98** |
|  | **Thiamine** | **-15.9342** | **Arg 463** | **H-Bond(Backbone)** | **1.72** |
|  |  |  | **Gly 196** | **H-Bond(Backbone)** | **2.22** |
|  | **Riboflavin** | **-15.1777** | **Glu 233** | **H-Bond(Sidechain)** | **1.66** |
|  |  |  |  | **H-Bond(Sidechain)** | **1.77** |
|  |  |  | **Arg 418** | **H-Bond(Backbone)** | **1.88** |
|  | **Spermidine** | **-12.6118** | **Gln 456** | **H-Bond(Sidechain)** | **2.19** |
|  |  |  | **Glu 230** | **H-Bond(Sidechain)** | **1.97** |
|  |  |  | **Gly 414** | **H-Bond(Backbone)** | **1.93** |
|  | **Retinol** | **-10.9987** | **Lys 199** | **H-Bond(Sidechain)** | **1.89** |
|  |  |  | **Gly 198** | **H-Bond(Backbone)** | **1.89** |
|  | **δ-Tocopherol** | **-1.1453** | **Lys 199** | **H-Bond(Backbone)** | **1.89** |
|  |  |  | **Arg 463** | **Pi- cation** | **5.22** |
|  |  |  |  | **Pi Pi stacking** | **4.56** |
|  |  |  | **Asn 416** | **H-Bond(Sidechain)** | **2.01** |
|  | **α-Tocopherol** | **-0.3838** | **Glu 233** | **H-Bond(Sidechain)** | **1.85** |
|  | **Tocomonoenol** | **-0.1126** | **Lys 201** | **H-Bond(Backbone)** | **1.89** |
|  |  |  |  | **H-Bond(Sidechain)** | **2.19** |
|  | **Phylloquinone** | **1.0276** | **Arg 202** | **H-Bond(Sidechain)** | **1.83** |

**Target- NS2B-NS3 (PDB ID- 3U1I)**

| **Sr. No.** | **Ligand** | **Dock Score** | **Interacting Residue** | **Bond Type** | **Bond distance** |
| --- | --- | --- | --- | --- | --- |
|  | **Phenylethyl alcohol** | **-16.748** | **Ser 135** | **H-Bond(Sidechain)** | **1.548** |
|  | **Ascorbic acid** | **-16.1999** | **Thr 134** | **H-Bond(Backbone)** | **1.776** |
|  |  |  | **Ser 135** | **H-Bond(Sidechain)** | **1.516** |
|  |  |  | **Tyr150** | **H-Bond(Sidechain)** | **2.016** |
|  | **23-hydroxytormentic acid** | **-15.3335** | **Thr A:83** | **H-Bond(Sidechain)** | **1.982** |
|  |  |  | **Asp A:81** | **H-Bond(Backbone)** | **1.989** |
|  |  |  |  | **H-Bond(Backbone)** | **2.237** |
|  | **Ethyl-3-hydroxybutyrate** | **-15.0731** | **Ser 135** | **H-Bond(Sidechain)** | **1.88** |
|  |  |  |  | **H-Bond(Backbone)** | **2.647** |
|  |  |  | **Thr 134** | **H-Bond(Backbone)** | **2.245** |
|  | **α-Terpineol** | **-13.5532** | **Ser 135** | **H-Bond(Backbone)** | **1.839** |
|  | **3-hydroxy-2-butanone** | **-12.5938** | **Ser 135** | **H-Bond(Backbone)** | **1.839** |
|  |  |  |  | **H-Bond(Sidechain)** | **1.389** |
|  |  |  | **Gly 133** | **H-Bond(Backbone)** | **2.121** |
|  |  |  | **Thr 134** | **H-Bond(Backbone)** | **2.141** |
|  | **Geraniol** | **-9.8528** | **Ser 135** | **H-Bond(Sidechain)** | **1.749** |
|  | **3-methyl-2-butanone** | **-7.9518** | **Tyr 161** | **H-Bond(Sidechain)** | **1.999** |
|  |  |  | **Gly 153** | **H-Bond(Backbone)** | **2.185** |
|  | **E-2-Hexenal** | **-5.4705** | **Tyr 161** | **H-Bond(Sidechain)** | **2.246** |
|  |  |  | **Gly 153** | **H-Bond(Backbone)** | **1.786** |
|  | **Kaempferol-3-rutinoside** | **-32.2516** | **Asp A:81** | **H-Bond(Sidechain)** | **1.856** |
|  |  |  |  | **H-Bond(Backbone)** | **2.408** |
|  |  |  |  | **H-Bond(Backbone)** | **1.811** |
|  |  |  | **Asn B:152** | **H-Bond(Sidechain)** | **2.509** |
|  |  |  | **Tyr B:161** | **H-Bond(Sidechain)** | **2.131** |
|  |  |  | **Gly B:151** | **H-Bond(Backbone)** | **1.819** |
|  |  |  | **Tyr B:150** | **H-Bond(Sidechain)** | **2.153** |
|  |  |  | **His B:51** | **Pi-Pi stacking** | **4.159** |
|  |  |  | **Val B:36** | **H-Bond(Backbone)** | **1.636** |
|  | **Riboflavin** | **-31.0863** | **Ser B:135** | **H-Bond(Backbone)** | **1.932** |
|  |  |  | **Lys B:131** | **H-Bond(Backbone)** | **2.232** |
|  |  |  | **Gly B:153** | **H-Bond(Backbone)** | **2.21** |
|  |  |  |  | **H-Bond(Backbone)** | **2.057** |
|  |  |  | **TyrB:161** | **H-Bond(Backbone)** | **2.071** |
|  |  |  | **Gly B:151** | **H-Bond(Backbone)** | **1.783** |
|  |  |  |  | **H-Bond(Backbone)** | **2.222** |
|  |  |  | **His B:51** | **Pi-Pi stacking** | **4.182** |
|  | **Kaemferol-3-rhamnoside** | **-28.4051** | **His 51** | **H-Bond(Backbone)** | **1.837** |
|  |  |  | **Lys 131** | **H-Bond(Backbone)** | **1.831** |
|  |  |  | **Gly 151** | **H-Bond(Backbone)** | **1.849** |
|  |  |  |  | **H-Bond(Backbone)** | **2.178** |
|  |  |  | **Tyr 161** | **H-Bond(Sidechain)** | **2.32** |
|  |  |  | **Gly 153** | **H-Bond(Backbone)** | **2.231** |
|  |  |  | **Asn 152** | **H-Bond(Sidechain)** | **2.251** |
|  | **Quercetin-3-glucoside** | **-28.1772** | **His 51** | **Pi- Pi stacking** | **4.13** |
|  |  |  | **Arg 54** | **H-Bond(Sidechain)** | **2.634** |
|  |  |  | **Asn 152** | **H-Bond(Sidechain)** | **2.149** |
|  |  |  | **Gly 153** | **H-Bond(Backbone)** | **1.883** |
|  |  |  | **Tyr 161** | **H-Bond(Sidechain)** | **1.73** |
|  |  |  | **Gly 151** | **H-Bond(Backbone)** | **1.870** |
|  |  |  | **Lys 131** | **H-Bond(Backbone)** | **2.374** |
|  |  |  | **Tyr 150** | **H-Bond(Sidechain)** | **2.075** |
|  |  |  | **Val 36** | **H-Bond(Backbone)** | **2.190** |
|  | **Chlorogenic acid** | **-25.997** | **Thr A:83** | **H-Bond(Sidechain)** | **1.866** |
|  |  |  | **Gly B:151** | **H-Bond(Backbone)** | **1.904** |
|  |  |  | **Phe B:130** | **H-Bond(Backbone)** | **2.440** |
|  |  |  | **Gly B:133** | **H-Bond(Backbone)** | **1.727** |
|  |  |  | **Ser B:135** | **H-Bond(Backbone)** | **2.186** |
|  | **Quinic acid** | **-25.6353** | **Lys 131** | **H-Bond(Backbone)** | **1.774** |
|  |  |  | **Phe 130** | **H-Bond(Backbone)** | **2.05** |
|  |  |  | **Ser 135** | **H-Bond(Backbone)** | **2.728** |
|  |  |  | **Gly 133** | **H-Bond(Backbone)** | **1.968** |
|  |  |  | **Thr 134** | **H-Bond(Backbone)** | **2.782** |
|  | **Epicatechin** | **-25.207** | **Ser 135** | **H-Bond(Sidechain)** | **1.558** |
|  |  |  | **Lys 131** | **H-Bond(Backbone)** | **1.632** |
|  |  |  | **Phe 130** | **H-Bond(Backbone)** | **1.769** |
|  |  |  | **Gly 133** | **H-Bond(Backbone)** | **2.125** |
|  | **Caffeic acid** | **-24.2597** | **Asp129** | **H-Bond(Sidechain)** | **1.417** |
|  |  |  |  | **H-Bond(Sidechain)** | **1.454** |
|  |  |  | **Tyr 161** | **Pi- Pi stacking** | **4.62** |
|  |  |  | **Gly 133** | **H-Bond(Backbone)** | **1.78** |
|  | **Protocatechuic acid** | **-23.4913** | **Phe 130** | **H-Bond(Backbone)** | **1.536** |
|  |  |  | **Gly 133** | **H-Bond(Backbone)** | **1.737** |
|  | **Shikimic acid** | **-23.4639** | **Lys 131** | **H-Bond(Backbone)** | **1.686** |
|  |  |  | **Phe 130** | **H-Bond(Backbone)** | **1.589** |
|  |  |  | **Gly 133** | **H-Bond(Backbone)** | **2.138** |
|  | **p-coumaric acid** | **-20.6049** | **Asp 129** | **H-Bond(Sidechain)** | **1.958** |
|  |  |  | **Gly 133** | **H-Bond(Backbone)** | **1.967** |
|  | **Serotonin** | **-20.1506** | **Ser 135** | **H-Bond(Sidechain)** | **2.162** |
|  |  |  | **Gly 133** | **H-Bond(Backbone)** | **2.208** |
|  |  |  | **Tyr 150** | **H-Bond(Sidechain)** | **1.88** |
|  |  |  | **Tyr 161** | **Pi cation** | **5.608** |
|  |  |  | **Asp 129** | **H-Bond(Sidechain)** | **1.880** |
|  | **Niacin** | **-18.205** | **Gly 133** | **H-Bond(Backbone)** | **1.840** |
|  | **Spermidine** | **-18.0467** | **Asp 129** | **H-Bond(Backbone)** | **2.076** |
|  |  |  |  | **H-Bond(Sidechain)** | **1.764** |
|  |  |  |  | **H-Bond(Sidechain)** | **1.705** |
|  |  |  | **Tyr 150** | **H-Bond(Sidechain)** | **2.14** |
|  |  |  | **Phe130** | **H-Bond(Backbone)** | **1.685** |
|  |  |  |  | **H-Bond(Backbone)** | **1.485** |
|  |  |  | **Tyr 161** | **Pi cation** | **6.072** |
|  | **Thiamine** | **-16.8927** | **Tyr 150** | **H-Bond(Sidechain)** | **1.647** |
|  |  |  | **Asp 129** | **H-Bond(Sidechain)** | **1.996** |
|  |  |  | **Gly 153** | **H-Bond(Backbone)** | **2.161** |
|  |  |  | **Tyr 161** | **H-Bond(Sidechain)** | **2.049** |
|  | **Malic acid** | **-15.9612** | **Thr 134** | **H-Bond(Backbone)** | **1.847** |
|  |  |  | **Gly 133** | **H-Bond(Backbone)** | **2.026** |
|  | **Putrescine** | **-15.061** | **Lys 131** | **H-Bond(Backbone)** | **2.007** |
|  |  |  | **Phe 130** | **H-Bond(Backbone)** | **1.468** |
|  |  |  | **Asp 129** | **H-Bond(Sidechain)** | **1.728** |
|  |  |  | **Tyr 150** | **H-Bond(Sidechain)** | **1.829** |
|  | **Retinol** | **-12.0656** | **Thr B:134** | **H-Bond(Backbone)** | **1.531** |
|  | **Phylloquinol** | **-11.8918** | **Gly 133** | **H-Bond(Backbone)** | **1.961** |
|  | **9-cis-neoxanthin** | **-8.0498** | **Val B:155** | **H-Bond(Backbone)** | **1.764** |
|  | **Tocomonoenol** | **-4.6901** | **Gly 133** | **H-Bond(Backbone)** | **2.174** |
|  |  |  | **Ser 135** | **H-Bond(Sidechain)** | **1.9** |
|  | **α- Tocopherol** | **-3.8813** | **Gly 133** | **H-Bond(Backbone)** | **1.983** |
|  |  |  | **Ser 135** | **H-Bond(Sidechain)** | **2.066** |
|  | **δ-Tocopherol** | **-2.8923** | **Gly 133** | **H-Bond(Backbone)** | **2.07** |
|  |  |  | **Ser 135** | **H-Bond(Sidechain)** | **1.899** |

**Target- NS5 SAM Binding pocket ( PDB ID- 5EHI)**

| **Sr. No.** | **Ligand** | **Dock Score** | **Interacting Residue** | **Bond Type** | **Bond distance** |
| --- | --- | --- | --- | --- | --- |
|  | **Quercetin -3-glucoside** | **-32.7547** | **Gly 148** | **H-Bond(Backbone)** | **1.99** |
|  |  |  | **Glu 111** | **H-Bond(Backbone)** | **2.28** |
|  |  |  | **Asp 131** | **H-Bond(Sidechain)** | **1.58** |
|  |  |  | **Gly 81** | **H-Bond(Backbone)** | **2.2** |
|  |  |  | **Lys 105** | **Pi-cation** | **6.13** |
|  |  |  |  | **H-Bond(Backbone)** | **1.61** |
|  |  |  | **Lys 130** | **H-Bond(Backbone)** | **1.73** |
|  |  |  | **Thr 104** | **H-Bond(Sidechain)** | **2.23** |
|  | **Kaempferol-3-rhamnoside** | **-31.1157** | **Gly 148** | **H-Bond(Backbone)** | **1.78** |
|  |  |  | **Thr 104** | **H-Bond(Sidechain)** | **1.87** |
|  |  |  | **Lys 105** | **H-Bond(Backbone)** | **2.13** |
|  |  |  | **Lys 130** | **H-Bond(Backbone)** | **2.11** |
|  |  |  | **Asp 131** | **H-Bond(Sidechain)** | **1.51** |
|  | **Epicatechin** | **-28.6107** | **Lys 130** | **H-Bond(Backbone)** | **1.78** |
|  |  |  | **Val 132** | **H-Bond(Backbone)** | **1.70,2.33** |
|  |  |  | **Asp 131** | **H-Bond(Sidechain)** | **2.29** |
|  |  |  | **Gly 148** | **H-Bond(Backbone)** | **1.81** |
|  |  |  | **Asp 146** | **H-Bond(Backbone)** | **2.21** |
|  |  |  | **Gly 81** | **H-Bond(Backbone)** | **1.81** |
|  | **Kaempferol-3-rutinoside** | **-24.9142** | **Glu 111** | **H-Bond(Backbone)** | **1.73** |
|  |  |  | **Thr 104** | **H-Bond(Sidechain)** | **2.20** |
|  |  |  | **Lys 130** | **H-Bond(Backbone)** | **2.13** |
|  |  |  | **Lys 105** | **H-Bond(Backbone)** | **2.09** |
|  |  |  |  | **Pi cation** | **6.35** |
|  | **Riboflavine** | **-24.7529** | **Gly 148** | **H-Bond(Backbone)** | **1.86** |
|  |  |  |  | **H-Bond(Backbone)** | **2.19** |
|  |  |  | **Asp 146** | **H-Bond(Sidechain)** | **1.92** |
|  |  |  |  | **H-Bond(Backbone)** | **2.26** |
|  |  |  | **Lys 105** | **H-Bond(Backbone)** | **2.22** |
|  | **Shikimic aicd** | **-24.4277** | **Asp 146** | **H-Bond(Sidechain)** | **2.10** |
|  |  |  | **Ser 56** | **H-Bond(Sidechain)** | **2.54** |
|  |  |  | **Cys 82** | **H-Bond(Backbone)** | **2.07** |
|  | **Caffeic acid** | **-23.7853** | **Lys 105** | **Pi cation** | **6.44** |
|  |  |  | **Val 132** | **H-Bond(Backbone)** | **1.93** |
|  |  |  | **Asp 131** | **H-Bond(Sidechain)** | **1.67** |
|  | **Chlorogenic acid** | **-23.5903** | **Glu 111** | **H-Bond(Backbone)** | **1.67** |
|  |  |  | **Gly 109** | **H-Bond(Backbone)** | **1.94** |
|  |  |  | **Lys 105** | **Pi cation** | **6.45** |
|  |  |  | **Lys 130** | **H-Bond(Backbone)** | **1.87** |
|  |  |  | **Val 132** | **H-Bond(Backbone)** | **1.51** |
|  | **Protocatechuic acid** | **-23.1145** | **G;y 148** | **H-Bond(Backbone)** | **1.87** |
|  |  |  | **Asp 146** | **H-Bond(Backbone)** | **1.75** |
|  |  |  | **Lys 105** | **H-Bond(Backbone)** | **2.11** |
|  |  |  | **Gly 81** | **H-Bond(Backbone)** | **1.95** |
|  | **Quinic acid** | **-22.3137** | **Asp 146** | **H-Bond(Sidechain)** | **1.66** |
|  |  |  | **Ser 56** | **H-Bond(Sidechain)** | **1.87** |
|  |  |  | **Gly 85** | **H-Bond(Backbone)** | **1.99** |
|  |  |  | **Gly 86** | **H-Bond(Backbone)** | **2.10** |
|  |  |  | **Trp 87** | **H-Bond(Backbone)** | **2.27** |
|  | **Malic acid** | **-21.4292** | **Gly 85** | **H-Bond(Backbone)** | **2.099** |
|  |  |  | **Gly 86** | **H-Bond(Backbone)** | **1.84** |
|  |  |  | **Trp 87** | **H-Bond(Backbone)** | **2.24** |
|  | **Spermidine** | **-19.67** | **Asp 146** | **Salt bridge** | **2.59** |
|  |  |  |  | **H-Bond(Sidechain)** | **1.95** |
|  |  |  | **Glu 216** | **Salt bridge** | **4.54** |
|  |  |  | **Trp 87** | **Pi cation** | **3.19** |
|  | **Serotonin** | **-19.5508** | **Hie 110** | **Pi Pi stacking** | **4.937** |
|  |  |  | **Lys 130** | **H-Bond(Backbone)** | **1.93** |
|  |  |  | **Val 132** | **H-Bond(Backbone)** | **1.86** |
|  |  |  | **Gly 81** | **H-Bond(Backbone)** | **2.02** |
|  |  |  | **Asp 146** | **H-Bond(Backbone)** | **1.78** |
|  | **p-coumaric acid** | **-18.3517** | **Lys 105** | **Pi cation** | **6.20** |
|  |  |  | **Asp 131** | **H-Bond(Sidechain)** | **1.86** |
|  | **Niacin** | **-17.7996** | **Gly 85** | **H-Bond(Backbone)** | **2.03** |
|  |  |  | **Gly 86** | **H-Bond(Backbone)** | **1.72** |
|  |  |  | **Trp 87** | **H-Bond(Backbone)** | **1.94** |
|  | **Thiamine** | **-16.4335** | **Val 132** | **H-Bond(Backbone)** | **1.83** |
|  |  |  | **Lys 130** | **H-Bond(Backbone)** | **1.94** |
|  |  |  | **Hie 110** | **Pi Pi stacking** | **5.13** |
|  |  |  | **Lys 105** | **Pi cation** | **6.31** |
|  |  |  | **Gly 148** | **H-Bond(Backbone)** | **1.86** |
|  | **Putrescine** | **-15.798** | **Gly 81** | **H-Bond(Backbone)** | **2.12** |
|  |  |  | **Trp 87** | **3 Pi cation** | **2.75, 6.05,6.17** |
|  |  |  | **Asp 146** | **H-Bond(Backbone)** | **1.90** |
|  |  |  |  | **H-Bond(Sidechain)** | **1.932** |
|  |  |  |  | **H-Bond(Sidechain)** | **1.76** |
|  | **Phylloquinone** | **-12.0475** | **Lys 105** | **Pi cation** | **6.45** |
|  |  |  |  | **H-Bond(Backbone)** | **1.94** |
|  | **Retinol** | **-11.8995** | **Gly 86** | **H-Bond(Backbone)** | **1.86** |
|  |  |  | **Lys 82** | **H-Bond(Backbone)** | **2.14** |
|  | **Tocomonoenol** | **-11.0019** | **Val 132** | **H-Bond(Backbone)** | **1.64** |
|  |  |  | **Asp 131** | **H-Bond(Sidechain)** | **1.898** |
|  |  |  | **Lys 105** | **Pi cation** | **6.16** |
|  | **α-Tocopherol** | **-8.7131** | **Val 132** | **H-Bond(Backbone)** | **1.63** |
|  |  |  | **Asp 131** | **H-Bond(Sidechain)** | **1.83** |
|  |  |  | **Lys 105** | **Pi cation** | **6.07** |
|  | **Δ-tocopherol** | **-7.1063** | **Lys 105** | **H-Bond(Backbone)** | **1.98** |
|  | **Ascorbic acid** | **-26.2121** | **Asp 146** | **H-Bond(Sidechain)** | **1.91** |
|  |  |  |  | **H-Bond(Sidechain)** | **1.84** |
|  |  |  | **Ser 56** | **H-Bond(Sidechain)** | **2.23** |
|  |  |  | **Gly 85** | **H-Bond(Backbone)** | **2.35** |
|  |  |  | **Gly 86** | **H-Bond(Backbone)** | **1.83** |
|  |  |  | **Trp 87** | **H-Bond(Backbone)** | **2.27** |
|  | **3-hydroxy-2-butanone** | **-13.5887** | **Lys 105** | **H-Bond(Backbone)** | **1.88** |
|  | **Phenylethyl alcohol** | **-13.1761** | **Lys 105** | **Pi cation** | **6.39** |
|  | **Ethyl-3-hydroxy** | **-13.0956** | **No interaction** | |  |
|  | **Α-Terpineol** | **-11.9623** | **Ser 56** | **H-Bond(Sidechain)** | **2.45** |
|  |  |  | **Cys 82** | **H-Bond(Backbone)** | **2.13** |
|  | **23-hydroxytormentic acid** | **-11.169** | **No interaction** | |  |
|  | **3-methyl-2-butanol** | **-9.2086** | **Gly 85** | **H-Bond(Backbone)** | **2.3** |
|  |  |  | **Gly86** | **H-Bond(Backbone)** | **1.89** |
|  | **Geraniol** | **-8.5761** | **Cys 82** | **H-Bond(Backbone)** | **2.03** |
|  |  |  | **Gly 86** | **H-Bond(Backbone)** | **1.62** |
|  | **2-E-hexenal** | **-3.9751** | **No interaction** | | **-** |

**Target- E glycoprotein ( PDB ID – 1OKE)**

| **Sr. No.** | **Ligand** | **Dock Score** | **Interacting Residue** | **Bond Type** | **Bond distance** |
| --- | --- | --- | --- | --- | --- |
|  | **Chlorogenic acid** | **-21.3989** | **Lys 128** | **H-Bond(Sidechain)** | **2.08** |
|  |  |  |  | **H-Bond(Sidechain)** | **1.73** |
|  |  |  |  | **Salt bridge** | **2.62** |
|  |  |  | **Gln 200** | **H-Bond(Sidechain)** | **1.85** |
|  |  |  | **Ala 50** | **H-Bond(Backbone)** | **2.36** |
|  |  |  | **Glu 49** | **H-Bond(Sidechain)** | **2.26** |
|  |  |  | **Thr 280** | **H-Bond(Backbone)** | **1.8** |
|  | **Serotonin** | **-20.906** | **Thr 48** | **H-Bond(Backbone)** | **1.63** |
|  |  |  | **Thr 280** | **H-Bond(Backbone)** | **1.62** |
|  | **Epicatechin** | **-19.2868** | **Thr 48** | **H-Bond(Backbone)** | **1.99** |
|  |  |  | **Phe 279** | **H-Bond(Backbone)** | **2.21** |
|  |  |  | **Thr 280** | **H-Bond(Backbone)** | **2.06** |
|  | **Quercetin-3-glucoside** | **-19.1458** | **Gln 271** | **H-Bond(Sidechain)** | **2.43** |
|  |  |  | **Thr 48** | **H-Bond(Backbone)** | **2.04** |
|  |  |  | **Glu 49** | **H-Bond(Sidechain)** | **1.82** |
|  |  |  | **Ala 50** | **H-Bond(Backbone)** | **1.48** |
|  | **Caffeic acid** | **-18.9577** | **Thr 48** | **H-Bond(Backbone)** | **1.92** |
|  |  |  |  | **H-Bond(Backbone)** | **1.83** |
|  |  |  | **Ala 50** | **H-Bond(Backbone)** | **1.94** |
|  |  |  | **Lys 128** | **Salt bridge** | **2.69** |
|  | **Kaemferol-3-rhamnoside** | **-18.2726** | **Ala 50** | **H-Bond(Backbone)** | **1.53** |
|  |  |  | **Gln 200** | **H-Bond(Sidechain)** | **2.15** |
|  |  |  |  | **H-Bond(Sidechain)** | **2.13** |
|  |  |  |  | **H-Bond(Sidechain)** | **1.98** |
|  |  |  | **Lys 128** | **H-Bond(Sidechain)** | **1.7** |
|  |  |  | **Thr 48** | **H-Bond(Backbone)** | **1.92** |
|  |  |  | **Gln 271** | **H-Bond(Sidechain)** | **2.3** |
|  |  |  | **Asp 203** | **H-Bond(Sidechain)** | **1.9** |
|  | **Shikimic acid** | **-16.8579** | **Lys 128** | **H-Bond(Sidechain)** | **1.83** |
|  |  |  |  | **H-Bond(Sidechain)** | **1.84** |
|  |  |  | **Gln 200** | **H-Bond(Sidechain)** | **2.17** |
|  |  |  | **Gln 49** | **H-Bond(Sidechain)** | **2.02** |
|  |  |  |  | **H-Bond(Backbone)** | **2.04** |
|  |  |  | **Ala 50** | **H-Bond(Backbone)** | **2.44** |
|  | **p-coumaric acid** | **-16.6234** | **Ala 50** | **H-Bond(Backbone)** | **2.14** |
|  |  |  | **Thr 48** | **H-Bond(Backbone)** | **1.9** |
|  | **Riboflavine** | **-16.0334** | **Ala 50** | **H-Bond(Backbone)** | **1.84** |
|  |  |  |  | **H-Bond(Backbone)** | **2.07** |
|  |  |  |  | **H-Bond(Backbone)** | **2.17** |
|  |  |  |  | **H-Bond(Backbone)** | **1.74** |
|  |  |  | **Glu 49** | **H-Bond(Sidechain)** | **1.91** |
|  |  |  | **Thr 48** | **H-Bond(Backbone)** | **1.80** |
|  | **Malic acid** | **-13.6405** | **Lys 128** | **Salt bridge** | **4.71** |
|  |  |  |  | **H-Bond(Sidechain)** | **2.00** |
|  |  |  |  | **H-Bond(Sidechain)** | **1.68** |
|  |  |  | **Ala 50** | **H-Bond(Backbone)** | **2.14** |
|  | **Quinic acid** | **-13.5931** | **Lys 28** | **Salt bridge** | **4.84** |
|  |  |  |  | **H-Bond(Sidechain)** | **2.02** |
|  |  |  | **Glu 49** | **H-Bond(Sidechain)** | **2.09** |
|  |  |  | **Ala 50** | **H-Bond(Backbone)** | **1.99** |
|  | **Protocatechuic acid** | **-13.4528** | **Thr 48** | **H-Bond(Backbone)** | **1.94** |
|  |  |  |  | **H-Bond(Backbone)** | **1.86** |
|  |  |  | **Gln 200** | **H-Bond(Sidechain)** | **1.87** |
|  | **Thiamine** | **-13.1064** | **Thr 48** | **H-Bond(Backbone)** | **2.22** |
|  |  |  | **Thr 280** | **H-Bond(Sidechain)** | **1.86** |
|  |  |  | **Gln 200** | **H-Bond(Sidechain)** | **1.88** |
|  | **Putrescine** | **-10.9586** | **Thr 280** | **H-Bond(Backbone)** | **1.99** |
|  |  |  | **His 282** | **H-Bond(Backbone)** | **1.49** |
|  |  |  | **Thr 189** | **H-Bond(Backbone)** | **1.65** |
|  | **Spermidine** | **-10.9007** | **Phe 279** | **H-Bond(Backbone)** | **2.24** |
|  |  |  | **Thr 280** | **H-Bond(Backbone)** | **1.76** |
|  |  |  | **His 282** | **H-Bond(Backbone)** | **1.80** |
|  |  |  | **Pro 187** | **H-Bond(Backbone)** | **1.99** |
|  |  |  | **Thr 189** | **H-Bond(Backbone)** | **1.39** |
|  | **Kaemferol -3-rutinoside** | **-10.3322** | **Lys 202** | **H-Bond(Backbone)** | **2.7** |
|  |  |  | **Asp 203** | **H-Bond(Sidechain)** | **1.89** |
|  |  |  |  | **H-Bond(Sidechain)** | **1.96** |
|  |  |  | **Ala 50** | **H-Bond(Backbone)** | **2.15** |
|  |  |  |  | **H-Bond(Backbone)** | **2.24** |
|  |  |  | **Thr 48** | **H-Bond(Backbone)** | **1.9** |
|  |  |  |  | **H-Bond(Backbone)** | **1.63** |
|  |  |  | **Lys 47** | **H-Bond(Sidechain)** | **2.37** |
|  | **Niacin** | **-10.1518** | **Leu 191** | **H-Bond(Backbone)** | **1.85** |
|  | **Retinol** | **-10.0719** | **Thr 189** | **H-Bond(Backbone)** | **1.68** |
|  | **Phylloquinone** | **-7.9925** | **Ala 50** | **H-Bond(Backbone)** | **2.04** |
|  |  |  | **Gln 271** | **H-Bond(Sidechain)** | **2.02** |
|  | **9’-cis-nioxanthin** | **-7.1578** | **Ala 50** | **H-Bond(Backbone)** | **1.64** |
|  |  |  | **Thr 48** | **H-Bond(Backbone)** | **1.76** |
|  | **Δ-Tocopherol** | **-2.1146** | **Gln 200** | **H-Bond(Sidechain)** | **2.09** |
|  |  |  | **Ala 50** | **H-Bond(Backbone)** | **2.18** |
|  | **Tocomonoenol** | **-0.6104** | **Gln 271** | **H-Bond(Sidechain)** | **2.14** |
|  |  |  | **Thr 48** | **H-Bond(Backbone)** | **1.77** |
|  |  |  | **Ala 50** | **H-Bond(Backbone)** | **1.99** |
|  | **α-Tocopherol** | **0.5078** | **Gln 271** | **H-Bond(Sidechain)** | **1.91** |
|  |  |  | **Thr 48** | **H-Bond(Backbone)** | **1.71** |
|  | **23-hydroxytormentic acid** | **-17.0119** | **Gln 52** | **H-Bond(Sidechain)** | **2.04** |
|  |  |  | **Lys 128** | **Salt bridge** | **2.46** |
|  |  |  | **Glu 49** | **H-Bond(Sidechain)** | **1.65** |
|  |  |  | **Thr 48** | **H-Bond(Backbone)** | **1.89** |
|  |  |  |  | **H-Bond(Backbone)** | **1.57** |
|  |  |  | **Ala 50** | **H-Bond(Backbone)** | **1.92** |
|  | **Ascorbic acid** | **-12.6046** | **Gly 281** | **H-Bond(Backbone)** | **2.00** |
|  |  |  | **Pro 187** | **H-Bond(Backbone)** | **2.06** |
|  |  |  | **Leu 191** | **H-Bond(Backbone)** | **2.19** |
|  | **Phenylethyl alcohol** | **-9.8679** | **Thr 48** | **H-Bond(Backbone)** | **2.21** |
|  | **Ethyl-3-hydroxybutyrate** | **-6.9925** | **Leu 191** | **H-Bond(Backbone)** | **1.61** |
|  |  |  | **His 282** | **H-Bond(Backbone)** | **1.82** |
|  | **3-hydroxy-2-butanone** | **-6.6292** | **Thr 280** | **H-Bond(Backbone)** | **2.01** |
|  |  |  | **Leu 191** | **H-Bond(Backbone)** | **1.98** |
|  | **α-Terpineol** | **-6.4605** | **Pro 187** | **H-Bond(Backbone)** | **1.77** |
|  | **Geraniol** | **-5.3029** | **Leu 191** | **H-Bond(Backbone)** | **2.02** |
|  |  |  | **Thr 189** | **H-Bond(Backbone)** | **2.12** |
|  | **3-methyl-2-butanone** | **-3.657** | **Leu 191** | **H-Bond(Backbone)** | **2.06** |
|  | **E-hexenal** | **-1.0222** | **No interaction** | | |

**Target- E glycoprotein (PDB ID 1OKE- Domain 3)**

| **Sr. No.** | **Ligand** | **Dock Score** | **Interacting Residue** | **Bond Type** | **Bond distance** |
| --- | --- | --- | --- | --- | --- |
|  | **Ascorbic acid** | **-12.9421** | **Phe 337** | **H-Bond(Backbone)** | **1.82** |
|  |  |  |  | **H-Bond(Backbone)** | **1.65** |
|  |  |  |  | **H-Bond(Backbone)** | **1.96** |
|  |  |  | **Ile 335** | **H-Bond(Backbone)** | **1.91** |
|  |  |  | **Asn 355** | **H-Bond(Backbone)** | **2.14** |
|  | **3-Hydroxy-2-butanone** | **-6.6471** | **Tyr 326** | **H-Bond(Sidechain)** | **1.89** |
|  |  |  | **Thr 303** | **H-Bond(Backbone)** | **1.9** |
|  | **3-Methyl-2-butanone** | **-3.9362** | **Tyr 326** | **H-Bond(Sidechain)** | **1.99** |
|  |  |  | **Thr 303** | **H-Bond(Backbone)** | **1.89** |
|  | **α-Terpineol** | **-3.3863** | **Phe 337** | **H-Bond(Backbone)** | **2.18** |
|  | **Phenylethyl alcohol** | **-2.5278** | **Asn 355** | **H-Bond(Sidechain)** | **1.93** |
|  | **Ethyl-3-hydroxybutyraldehyde** | **-2.1984** | **Phe 337** | **H-Bond(Backbone)** | **1.9** |
|  |  |  | **Ile 335** | **H-Bond(Backbone)** | **1.92** |
|  | **Geraniol** | **-2.0297** | **Phe 337** | **H-Bond(Backbone)** | **1.97** |
|  | **E-hexenal** | **-0.4072** | **Phe 337** | **H-Bond(Backbone)** | **1.89** |
|  | **23-hydroxytormentic acid** | **0.8474** | **Cys 302** | **H-Bond(Backbone)** | **2.35** |
|  |  |  | **Gln 386** | **H-Bond(Sidechain)** | **1.89** |
|  | **Epicatechin** | **-20.691** | **Ile 335** | **H-Bond(Backbone)** | **1.73** |
|  |  |  |  | **H-Bond(Backbone)** | **1.78** |
|  |  |  | **Phe 337** | **H-Bond(Backbone)** | **2.20** |
|  |  |  | **Leu 351** | **H-Bond(Backbone)** | **1.89** |
|  | **Kaemferol-3-rhamnoside** | **-14.6036** | **Phe 337** | **H-Bond(Backbone)** | **2.08** |
|  |  |  | **Gly 381** | **H-Bond(Backbone)** | **1.90** |
|  | **Protocatechuic acid** | **-14.4177** | **Phe 337** | **H-Bond(Backbone)** | **1.93** |
|  |  |  | **Pro 356** | **H-Bond(Backbone)** | **1.93** |
|  |  |  | **Asn 355** | **H-Bond(Sidechain)** | **1.88** |
|  | **Serotonin** | **-14.2435** | **Glu 338** | **Salt bridge** | **3.77** |
|  |  |  | **Ile 335** | **H-Bond(Backbone)** | **1.54** |
|  |  |  | **Asn 355** | **H-Bond(Sidechain)** | **2.17** |
|  | **Shikimic acid** | **-13.6214** | **Asn 355** | **H-Bond(Sidechain)** | **2.06** |
|  |  |  |  | **H-Bond(Sidechain)** | **2.08** |
|  |  |  | **Phe 337** | **H-Bond(Backbone)** | **2.01** |
|  | **Quercetin-3-glucoside** | **-13.1589** | **Asn 355** | **H-Bond(Backbone)** | **2.13** |
|  |  |  | **Pro 356** | **H-Bond(Backbone)** | **2.13** |
|  |  |  | **Phe 337** | **H-Bond(Backbone)** | **1.84** |
|  |  |  | **Glu 338** | **H-Bond(Sidechain)** | **2.11** |
|  |  |  | **Leu 351** | **H-Bond(Backbone)** | **2.09** |
|  | **p-coumaric acid** | **-12.6997** | **Phe 337** | **H-Bond(Backbone)** | **1.89** |
|  |  |  | **Lys 344** | **Salt bridge** | **4.77** |
|  |  |  | **Glu 383** | **H-Bond(Backbone)** | **2.11** |
|  | **Caffeic acid** | **-11.93** | **Leu 351** | **H-Bond(Backbone)** | **2.00** |
|  |  |  | **Ile 335** | **H-Bond(Backbone)** | **1.91** |
|  |  |  | **Phe 337** | **H-Bond(Backbone)** | **1.91** |
|  | **Riboflavin** | **-11.618** | **Phe 337** | **H-Bond(Backbone)** | **2.56** |
|  |  |  | **Glu 338** | **H-Bond(Sidechain)** | **2.19** |
|  |  |  |  | **H-Bond(Sidechain)** | **1.76** |
|  |  |  |  | **H-Bond(Sidechain)** | **1.67** |
|  |  |  | **Gly 381** | **H-Bond(Backbone)** | **1.56** |
|  |  |  | **Glu 383** | **H-Bond(Backbone)** | **2.40** |
|  | **Chlorogenic acid** | **-11.2154** | **Leu 351** | **H-Bond(Backbone)** | **2.17** |
|  |  |  | **Glu 338** | **H-Bond(Sidechain)** | **2.08** |
|  |  |  | **Ile 339** | **H-Bond(Backbone)** | **2.77** |
|  |  |  | **Asn 355** | **H-Bond(Sidechain)** | **1.56** |
|  |  |  |  | **H-Bond(Sidechain)** | **1.61** |
|  | **Quinic acid** | **-11.1375** | **Asn 355** | **H-Bond(Backbone)** | **2.06** |
|  |  |  | **Phe 337** | **H-Bond(Backbone)** | **1.88** |
|  |  |  | **Ile 335** | **H-Bond(Backbone)** | **1.8** |
|  | **9’-cis-neoxanthin** | **-10.3474** | **Asn 355** | **H-Bond(Backbone)** | **1.91** |
|  |  |  |  | **H-Bond(Sidechain)** | **2.12** |
|  |  |  | **Gln 386** | **H-Bond(Sidechain)** | **2.13** |
|  |  |  | **Glu 383** | **H-Bond(Backbone)** | **1.83** |
|  | **Spermidine** | **-9.5556** | **Ile 335** | **H-Bond(Backbone)** | **2.18** |
|  |  |  | **Phe 337** | **H-Bond(Backbone)** | **2.2** |
|  |  |  | **Ile379** | **H-Bond(Backbone)** | **1.99** |
|  |  |  | **Gly 381** | **H-Bond(Backbone)** | **2.3** |
|  |  |  |  | **H-Bond(Sidechain)** | **1.67** |
|  | **Thiamine** | **-9.4148** | **Leu 351** | **H-Bond(Backbone)** | **1.85** |
|  |  |  | **Ile 335** | **H-Bond(Backbone)** | **2.13** |
|  |  |  | **Phe 337** | **H-Bond(Backbone)** | **1.91** |
|  | **Kaempferol -3-rutinoside** | **-9.2122** | **Gly 349** | **H-Bond(Backbone)** | **2.22** |
|  |  |  | **Glu 338** | **H-Bond(Sidechain)** | **2.07** |
|  |  |  |  | **H-Bond(Sidechain)** | **2.23** |
|  |  |  | **Phe 337** | **H-Bond(Backbone)** | **1.82** |
|  |  |  | **Pro 356** | **H-Bond(Backbone)** | **2.18** |
|  |  |  | **Asn 355** | **H-Bond(Backbone)** | **2.12** |
|  | **Niacin** | **-9.0871** | **Gly 304** | **H-Bond(Backbone)** | **2.48** |
|  |  |  | **Thr 303** | **H-Bond(Backbone)** | **2.03** |
|  | **Malic acid** | **-8.2719** | **Asn 355** | **H-Bond(Backbone)** | **2.13** |
|  |  |  | **Ile 335** | **H-Bond(Backbone)** | **1.77** |
|  |  |  | **Phe 337** | **H-Bond(Backbone)** | **2.14** |
|  | **Putrescine** | **-7.4843** | **Gly 381** | **H-Bond(Backbone)** | **1.59** |
|  |  |  | **Glu 338** | **H-Bond(Sidechain)** | **1.62** |
|  | **Retinol** | **-4.6866** | **Gly 381** | **H-Bond(Backbone)** | **1.90** |
|  | **Phylloquinone** | **2.3927** | **Glu 383** | **H-Bond(Backbone)** | **1.88** |
|  | **Tocomonoenol** | **2.7355** | **Phe 337** | **H-Bond(Backbone)** | **1.55** |
|  | **α-Tocopherol** | **3.6835** | **Phe 337** | **H-Bond(Backbone)** | **1.55** |
|  | **δ-Tocopherol** | **4.1096** | **Ile 335** | **H-Bond(Backbone)** | **1.73** |

**Target- Stem Domain (Model)**

| **Sr. No.** | **Ligand** | **Dock Score** | **Interacting Residue** | **Bond Type** | **Bond distance** |
| --- | --- | --- | --- | --- | --- |
|  | **Phenylethylalcohol** | **-10.6223** | **Arg 691** | **H-Bond(Sidechain)** | **2.19** |
|  |  |  | **Trp 700** | **Pi Pi stacking** | **4.83** |
|  | **Ascorbic acid** | **-10.1678** | **Arg 691** | **H-Bond(Sidechain)** | **1.98, 2.01, 1.98** |
|  |  |  | **Gly 688** | **H-Bond(Backbone)** | **2.13** |
|  |  |  | **Ala 699** | **H-Bond(Backbone)** | **1.71** |
|  | **Α-Terpineol** | **-7.2942** | **Arg 691** | **H-Bond(Sidechain)** | **2.11** |
|  | **2- E-Hexenal** | **-6.863** | **Arg 691** | **H-Bond(Sidechain)** | **2.21** |
|  | **Ethyl-3-hydroxybutyrate** | **-5.5671** | **Arg 691** | **H-Bond(Sidechain)** | **2.14** |
|  |  |  | **Ala 699** | **H-Bond(Backbone)** | **1.84** |
|  | **3-methyl-2-butanone** | **-5.5655** | **Arg 691** | **H-Bond(Sidechain)** | **1.84** |
|  | **3-hydroxy-2-butanone** | **-5.324** | **Arg 691** | **H-Bond(Sidechain)** | **1.84** |
|  |  |  | **Ala 699** | **H-Bond(Backbone)** | **2.27** |
|  | **Geraniol** | **-4.6198** | **Gly 688** | **H-Bond(Backbone)** | **2.15** |
|  | **23-hydroxytormentic acid** | **-0.83** | **Phe 702** | **H-Bond(Backbone)** | **1.89** |
|  |  |  | **Phe 291** | **H-Bond(Backbone)** | **1.99** |
|  | **Prtocatechuic acid** | **-19.4906** | **Arg 691** | **H-Bond(Sidechain)** | **1.89** |
|  |  |  |  | **H-Bond(Sidechain)** | **1.65** |
|  |  |  |  | **Salt bridge** | **2.61** |
|  |  |  | **Phe 702** | **H-Bond(Backbone)** | **1.80** |
|  | **Shikimic acid** | **-18.5522** | **Arg 691** | **H-Bond(Sidechain)** | **1.81** |
|  |  |  |  | **Salt bridge** | **2.88** |
|  |  |  | **Phe 702** | **H-Bond(Backbone)** | **2.12** |
|  | **p-coumaric acid** | **-18.4392** | **Arg 691** | **H-Bond(Sidechain)** | **2.03** |
|  |  |  |  | **H-Bond(Sidechain)** | **1.52** |
|  | **Caffeic acid** | **-17.9774** | **Arg 691** | **H-Bond(Sidechain)** | **1.74** |
|  |  |  |  | **Salt bridge** | **2.85** |
|  |  |  | **Trp 700** | **H-Bond(Backbone)** | **1.79** |
|  | **alic acid** | **-17.3804** | **Arg 691** | **H-Bond(Sidechain)** | **1.94** |
|  |  |  |  | **H-Bond(Sidechain)** | **1.98** |
|  |  |  |  | **Salt bridge** | **4.08** |
|  | **Niacin** | **-16.3108** | **Arg 691** | **Salt bridge** | **4.69** |
|  |  |  |  | **H-Bond(Sidechain)** | **2.29** |
|  | **Kaempferol-3-rhamnoside** | **-16.0332** | **Trp 700** | **Pi- Pi stacking** | **4.68** |
|  |  |  | **Arg 691** | **H-Bond(Sidechain)** | **1.86** |
|  |  |  |  | **H-Bond(Sidechain)** | **2.07** |
|  |  |  | **Gly 688** | **H-Bond(Backbone)** | **1.70** |
|  | **Quinic acid** | **-14.9511** | **Arg 691** | **H-Bond(Sidechain)** | **1.89** |
|  |  |  |  | **H-Bond(Sidechain)** | **1.78** |
|  |  |  | **Phe 702** | **H-Bond(Backbone)** | **1.61** |
|  |  |  |  | **H-Bond(Backbone)** | **2.18** |
|  | **Epicatechin** | **-13.9125** | **Phe 702** | **H-Bond(Backbone)** | **1.73** |
|  |  |  | **Arg 691** | **H-Bond(Sidechain)** | **1.77** |
|  |  |  | **Gly 688** | **H-Bond(Backbone)** | **2.06** |
|  | **Serotonin** | **-13.8514** | **Gly 688** | **H-Bond(Backbone)** | **2.14** |
|  |  |  | **Phe 702** | **H-Bond(Backbone)** | **1.97** |
|  | **Thiamine** | **-9.6219** | **Arg 691** | **H-Bond(Sidechain)** | **1.95** |
|  |  |  | **Ala 699** | **H-Bond(Backbone)** | **1.79** |
|  |  |  | **Trp 700** | **Pi Pi stacking** | **4.29** |
|  | **Riboflavin** | **-9.2153** | **Trp 700** | **H-Bond(Backbone)** | **2.28** |
|  |  |  |  | **H-Bond(Backbone)** | **1.88** |
|  |  |  | **Arg 691** | **H-Bond(Sidechain)** | **1.98** |
|  | **Chlorogenic acid** | **-8.3285** | **Arg 691** | **H-Bond(Sidechain)** | **1.85** |
|  |  |  |  | **H-Bond(Sidechain)** | **1.74** |
|  | **Quercetin-3-glucoside** | **-5.9642** | **Arg 691** | **Pi Pi stacking** | **4.75** |
|  |  |  |  | **H-Bond(Sidechain)** | **2.71** |
|  |  |  | **Phe 702** | **H-Bond(Backbone)** | **1.77** |
|  |  |  | **Gly 688** | **H-Bond(Backbone)** | **2.11** |
|  | **Retinol** | **-5.6339** |  | **No interaction** |  |
|  | **Spermidine** | **-4.2717** | **Ala 699** | **H-Bond(Backbone)** | **2.15** |
|  |  |  | **Gly 688** | **H-Bond(Backbone)** | **1.93** |
|  |  |  | **Phe 702** | **H-Bond(Backbone)** | **2.08** |
|  | **Phylloquinone** | **-2.069** | **Trp 700** | **Pi Pi stacking** | **4.99** |
|  |  |  | **Arg 691** | **H-Bond(Sidechain)** | **1.72** |
|  | **Putrescine** | **-0.9844** | **Gly 688** | **H-Bond(Backbone)** | **1.85** |
|  |  |  | **Met 692** | **H-Bond(Backbone)** | **2.5** |
|  |  |  | **Ala 699** | **H-Bond(Backbone)** | **1.44** |
|  | **Tocomonoenol** | **-0.5417** | **Arg 691** | **H-Bond(Sidechain)** | **1.81** |
|  | **α-tocopherol** | **0.5449** | **Arg 691** | **H-Bond(Sidechain)** | **1.80** |
|  | **δ-tocopherol** | **2.0167** | **Gly 688** | **H-Bond(Backbone)** | **1.82** |

**Target- NS5 RdRp ( PDB ID- 5HMZ)**

| **Sr. No.** | **Ligand** | **Dock Score** | **Interacting Residue** | **Bond Type** | **Bond distance** |
| --- | --- | --- | --- | --- | --- |
|  | **Caffeic acid** | **-24.8427** | **His 798** | **H-Bond(Backbone)** | **2.07** |
|  |  |  | **Ser 796** | **H-Bond(Sidechain)** | **2.3** |
|  |  |  | **Arg 729** | **Salt bridge** | **3.74** |
|  | **Epicatechin** | **-24.3502** | **His 798** | **H-Bond(Backbone)** | **2.33** |
|  |  |  | **Thr 794** | **H-Bond(Sidechain)** | **2.08** |
|  |  |  | **Ser 710** | **H-Bond(Sidechain)** | **1.95** |
|  | **Chlorogenic acid** | **-23.9068** | **Leu 511** | **H-Bond(Backbone)** | **2.43** |
|  |  |  | **Arg 729** | **H-Bond(Sidechain)** | **2.73** |
|  |  |  |  | **H-Bond(Sidechain)** | **1.99** |
|  |  |  |  | **H-Bond(Sidechain)** | **1.97** |
|  |  |  | **Ser 710** | **H-Bond(Sidechain)** | **2.18** |
|  | **Quinic acid** | **-22.7297** | **Arg 729** | **H-Bond(Sidechain)** | **2.01** |
|  |  |  |  | **H-Bond(Sidechain)** | **1.85** |
|  |  |  | **Trp 795** | **H-Bond(Backbone)** | **2.13** |
|  | **Kaemferol-3-rhamnoside** | **-22.0444** | **Thr 794** | **H-Bond(Sidechain)** | **1.74** |
|  |  |  | **Tyr 766** | **H-Bond(Sidechain)** | **2.37** |
|  |  |  | **Thr 793** | **H-Bond(Backbone)** | **1.97** |
|  |  |  | **Arg 729** | **Pi cation** | **5.71** |
|  |  |  | **Ser 710** | **H-Bond(Sidechain)** | **2.29** |
|  | **Malic acid** | **-21.2725** | **Arg 729** | **H-Bond(Sidechain)** | **1.79** |
|  |  |  |  | **Salt bridge** | **3.98** |
|  |  |  | **Arg 737** | **H-Bond(Sidechain)** | **1.90** |
|  |  |  |  | **H-Bond(Sidechain)** | **1.84** |
|  | **Niacin** | **-19.7243** | **Arg 729** | **H-Bond(Sidechain)** | **2.07** |
|  | **Shikimic acid** | **-19.5305** | **Arg 729** | **H-Bond(Sidechain)** | **1.76** |
|  |  |  |  | **H-Bond(Sidechain)** | **2.14** |
|  |  |  | **Arg 737** | **Salt bridge** | **4.72** |
|  |  |  | **Thr 794** | **H-Bond(Sidechain)** | **1.92** |
|  |  |  | **Thr 793** | **H-Bond(Backbone)** | **1.50** |
|  | **p-coumaric acid** | **-19.2641** | **Arg 729** | **H-Bond(Sidechain)** | **1.74** |
|  |  |  |  | **H-Bond(Sidechain)** | **1.91** |
|  |  |  | **His 798** | **H-Bond(Backbone)** | **1.97** |
|  | **Protocatechuic acid** | **-19.0975** | **Thr 794** | **H-Bond(Sidechain)** | **1.60** |
|  |  |  | **Arg 729** | **H-Bond(Sidechain)** | **1.48** |
|  |  |  |  | **H-Bond(Sidechain)** | **1.97** |
|  | **Serotonin** | **-18.3588** | **His 801** | **Pi cation** | **6.28** |
|  |  |  | **Gln 802** | **H-Bond(Sidechain)** | **1.98** |
|  |  |  | **Ser 796** | **H-Bond(Sidechain)** | **2.11** |
|  | **Riboflavin** | **-13.5237** | **Cys 709** | **H-Bond(Backbone)** | **1.89** |
|  |  |  | **Arg 729** | **H-Bond(Sidechain)** | **1.94** |
|  |  |  | **Thr 794** | **H-Bond(Sidechain)** | **1.73** |
|  |  |  | **Ile 797** | **H-Bond(Backbone)** | **2.08** |
|  | **Quercetin -3-glucoside** | **-13.3811** | **Arg 729** | **H-Bond(Sidechain)** | **2.05** |
|  |  |  | **Tyr 766** | **H-Bond(Sidechain)** | **2.05** |
|  |  |  | **His 798** | **H-Bond(Backbone)** | **2.25** |
|  |  |  | **Ser 796** | **H-Bond(Sidechain)** | **1.96** |
|  | **Thiamine** | **-11.9795** | **Tyr 766** | **H-Bond(Sidechain)** | **1.99** |
|  |  |  | **Ser 796** | **H-Bond(Sidechain)** | **2.27** |
|  | **Retinol** | **-10.1994** | **His 798** | **H-Bond(Backbone)** | **2.15** |
|  |  |  | **His 800** | **H-Bond(Backbone)** | **1.95** |
|  | **Putrescine** | **-8.3171** | **Trp 795** | **H-Bond(Backbone)** | **1.72** |
|  |  |  | **Ile 797** | **H-Bond(Backbone)** | **1.79** |
|  |  |  | **Ser 796** | **H-Bond(Backbone)** | **1.9** |
|  |  |  | **Ala 799** | **H-Bond(Backbone)** | **1.58** |
|  | **δ-tocopherol** | **-4.7557** | **Tyr 766** | **H-Bond(Sidechain)** | **1.9** |
|  | **Phylloquinone** | **-4.5419** | **Ser 796** | **H-Bond(Sidechain)** | **2.17** |
|  | **Spermidine** | **-4.4418** | **Thr 793** | **H-Bond(Backbone)** | **1.86** |
|  |  |  | **Thr 794** | **H-Bond(Sidechain)** | **1.68** |
|  |  |  | **Cys 709** | **H-Bond(Backbone)** | **1.73** |
|  | **Tocomonoenol** | **-0.4368** | **Arg 729** | **Pi Pi stacking** | **4.77** |
|  |  |  | **His 711** | **Pi Pi stacking** | **5.42** |
|  | **α-tocopherol** | **-0.2829** | **Arg 729** | **Pi Pi stacking** | **5.48** |
|  |  |  | **His 711** | **Pi Pi stacking** | **4.84** |
|  | **Ascorbic acid** | **-14.8936** | **Arg 729** | **H-Bond(Sidechain)** | **2.8** |
|  |  |  |  | **H-Bond(Sidechain)** | **1.92** |
|  |  |  | **Tyr 766** | **H-Bond(Sidechain)** | **2.00** |
|  |  |  |  | **H-Bond(Sidechain)** | **1.77** |
|  |  |  | **Thr 794** | **H-Bond(Sidechain)** | **1.76** |
|  |  |  | **Ser 796** | **H-Bond(Sidechain)** | **2.20** |
|  | **3-hydorxy-2-butanone** | **-10.7201** | **Thr 794** | **H-Bond(Sidechain)** | **1.98** |
|  | **Phenylethyl alcohol** | **-8.7066** | **His 711** | **H-Bond(Backbone)** | **2.45** |
|  |  |  | **Arg 729** | **H-Bond(Sidechain)** | **2.04** |
|  | **Ethyl-3-hydroxybutyrate** | **-7.7791** | **Arg 729** | **H-Bond(Sidechain)** | **2.07** |
|  |  |  | **Thr 794** | **H-Bond(Sidechain)** | **1.69** |
|  | **Geraniol** | **-6.8477** | **His 798** | **H-Bond(Backbone)** | **2.13** |
|  | **α-terpineol** | **-4.965** | **Arg 729** | **H-Bond(Sidechain)** | **2.07** |
|  | **3-methyl-2-butanol** | **-4.5916** | **No interaction** | | |
|  | **2-E-hexenal** | **-3.2711** | **Arg 729** | **H-Bond(Sidechain)** | **2.15** |
